# Metabolic syndrome and inflammation in adipose tissue occur at different times in animals submitted to a high-sugar/fat diet

**DOI:** 10.1017/jns.2017.42

**Published:** 2017-08-21

**Authors:** Fabiane Valentini Francisqueti, André Ferreira Nascimento, Igor Otávio Minatel, Marcos Correa Dias, Renata de Azevedo Melo Luvizotto, Carolina Berchieri-Ronchi, Ana Lúcia A. Ferreira, Camila Renata Corrêa

**Affiliations:** 1 São Paulo State University (UNESP), Botucatu Medical School, Botucatu, São Paulo, Brazil; 2 Institute of Health Sciences, Federal University of Mato Grosso (UFMT), Sinop, Mato Grosso, Brazil; 3 São Paulo State University, Institute of Bioscience, Botucatu, São Paulo, Brazil

**Keywords:** Obesity, Adipocytes, Inflammation, Metabolic syndrome, C, control diet, C24, control diet for 24 weeks, HOMA-IR, homeostasis model assessment, HSF, high-sugar/fat diet, HSF6, high-sugar/fat diet for 6 weeks, HSF12, high-sugar/fat diet for 12 weeks, HSF24, high-sugar/fat diet for 24 weeks, MS, metabolic syndrome, TLR-4, Toll-like receptor-4

## Abstract

Obesity is associated with low-grade inflammation, triggered in adipose tissue, which may occur due to an excess of SFA from the diet that can be recognised by Toll-like receptor-4. This condition is involved in the development of components of the metabolic syndrome associated with obesity, especially insulin resistance. The aim of the study was to evaluate the manifestation of the metabolic syndrome and adipose tissue inflammation as a function of the period of time in which rats were submitted to a high-sugar/fat diet (HSF). Male Wistar rats were divided into six groups to receive the control diet (C) or the HSF for 6, 12 or 24 weeks. HSF increased the adiposity index in all HSF groups compared with the C group. HSF was associated with higher plasma TAG, glucose, insulin and leptin levels. Homeostasis model assessment increased in HSF compared with C rats at 24 weeks. Both TNF-α and IL-6 were elevated in the epididymal adipose tissue of HSF rats at 24 weeks compared with HSF at 6 weeks and C at 24 weeks. Only the HSF group at 24 weeks showed increased expression of both Toll-like receptor-4 and NF-κB. More inflammatory cells were found in the HSF group at 24 weeks. We can conclude that the metabolic syndrome occurs independently of the inflammatory response in adipose tissue and that inflammation is associated with hypertrophy of adipocytes, which varies according to duration of exposure to the HSF.

The prevalence of obesity has increased strikingly during the past three decades, particularly among minorities and socio-economically disadvantaged populations around the world^(^^1^^–^^3^^)^. The main factor that leads to this condition is overnutrition, especially when characterised by the excessive intake of carbohydrates and fat^(^^4^^–^^9^^)^, which can trigger the metabolic syndrome (MS) defined as a constellation of metabolic abnormalities for CVD and diabetes. A consensus agreement by the International Diabetes Federation and the American Heart Association/National Heart, Lung and Blood Institute identifies the criteria of the MS as abdominal obesity, reduced HDL, elevated TAG, glucose intolerance and hypertension; a diagnosis requires any three of these five criteria^(^^10^^)^.

The primary cause of the MS appears to be increased adiposity associated with insulin resistance^(^^11^^,^^12^^)^. Furthermore, there is a strong relationship between obesity and inflammation^(^^13^^)^, since hyperadiposity produces adipokines, such as leptin, adiponectin and resistin, as well as proinflammatory cytokines such as IL-6, TNF-α and plasminogen activator inhibitor type 1, which are all involved in proinflammatory and prothrombotic responses^(^^14^^)^.

In obesity, inflammation that is triggered in adipose tissue may occur due to the excess of SFA in the diet. These fatty acids can be recognised by Toll-like receptor-4 (TLR-4), which is expressed by adipocytes and macrophages, leading to activation of NF-κB^(^^15^^–^^18^^)^, stimulating the production of chemokines and proinflammatory cytokines^(^^19^^)^, as well as attracting immune cells from the circulation into the adipose tissue^(^^16^^)^. TLR-4 is a cell surface receptor that generates innate immune responses to pathogens by inducing signalling cascades of kinase and transcription factor activation, leading to the generation of proinflammatory cytokines, chemokines, eicosanoids and reactive oxygen species, all of which are effectors of innate immunity.

Thus, we can propose that excessive sugar and fat intake are factors that lead to inflammation in adipose tissue. Several studies have shown that inflammation is involved in the development of components of the MS associated with obesity, especially insulin resistance^(^^20^^–^^22^^)^. However, few experimental studies that have emphasised the role of diet, obesity and inflammation evaluated the participation of the TLR-4 pathway as a function of time^(^^23^^–^^28^^)^. Therefore, additional studies are needed to characterise the inflammatory response in adipose tissue and insulin resistance as a function of time. Thus, the aim of the present study was to evaluate the manifestation of the MS and inflammation adipose tissue as a function of time in rats submitted to a high-sugar/fat diet (HSF).

## Materials and methods

### Animals and experimental protocol

The experimental protocol was approved by the local Ethical Committee for Animal Research of the University of Sao Paulo State University (permit number PE-47/2011). Male Wistar rats (10 weeks old, ±350 g) from the Animal Center of Botucatu Medical School, Sao Paulo State University (UNESP, Botucatu, SP, Brazil), were assigned to either a commercial chow diet (control diet; C; 12 % energy from fat) or an HSF (49·7 % energy from fat) with sucrose in the drinking water (300 g/l) for a period of 6, 12 or 24 weeks (C6, HSF6, C12, HSF12, C24, HSF24). The diet-induced obesity model was adapted from our previous study^(^^29^^)^ and it has been published previously^(^^30^^)^, which was used to mimic obesity from Western occidental dietary habits.

Rats were housed in individual cages in the animal facility at the Internal Medicine Experimental Laboratory, Botucatu Medical School, UNESP, under controlled ambient temperature (22–26°C) and lighting (12 h light–12 h dark) conditions. Dietary and water consumption was measured daily, and body weight was assessed weekly. Energy intake was calculated according to the formula: energy intake (kJ/d) = food consumption (g) × dietary energy (kJ/g). For the animals that received sucrose in drinking water (30 %), the energy intake was calculated according to the formula: volume consumed (ml) × 0·3 (equivalent to 30 % sucrose) × 16·7 (kJ per g of carbohydrate) + energy values offered by feeding (food consumption (g) × dietary energy (kJ/g)).

The animals were killed by decapitation after anaesthesia with sodium pentobarbital Q4 (50 mg/kg, intraperitoneal injection) and all efforts were made to minimise suffering. Blood from fasted animals was collected in tubes containing EDTA and centrifuged at 3500 rpm and the plasma was collected for analysis. Epididymal adipose tissue was selected for analysis because of its similar inflammatory patterns to visceral fat^(^^31^^)^.

### Adiposity index

The adiposity index was used as an indicator of obesity because it enables the precise evaluation of body fat percentage. Epididymal, retroperitoneal and visceral fat deposits were dissected from the rats. The sum of the fat deposits, normalised by body weight, was calculated to obtain the adiposity index: ((epididymal + retroperitoneal + visceral)/body weight) × 100^(^^5^^,^^32^^)^.

### Plasma analysis

#### Biochemical

After 12 h of overnight fasting, plasma analysis were carried out. An enzymic colorimetric kit was used to measure glucose (Bioclin^®^; Belo Horizonte), TAG (Bioclin^®^; Belo Horizonte) and NEFA (WAKO^®^; HR Series NEFA-HR^(^^2^^)^). Spectrophotometry was performed with the Chemistry Analyser BS 200 automatic spectrophotometer (Mindray Medical International Ltd).

#### Insulin resistance

Insulin resistance was determined using the index of homeostasis model assessment (HOMA-IR) using the following formula^(^^33^^)^: HOMA-IR = fasting insulin (μU/ml) × fasting glucose (mmol/l)/22·5.

#### Hormones and inflammatory cytokines

Plasma levels of insulin, leptin, adiponectin, TNF-α and IL-6 were measured by ELISA. Insulin, leptin and adiponectin ELISA kits were purchased from Millipore Corporation and TNF-α and IL-6 ELISA kits were purchased from R&D Systems. A microplate spectrophotometer reader (SpectraMax 190; Molecular Devices) was used according to the manufacturer's instructions.

### Analysis of epididymal adipose tissue

#### Adipokine levels

Epididymal adipose tissue (400 mg) was triturated with 2 ml of PBS (pH 7·4) and then centrifuged at 3000 rpm and 4°C for 10 min. Using the supernatants, TNF-α and IL-6 were measured using commercial ELISA kits (R&D Systems) according to the manufacturer's instructions. The results were normalised to protein amounts of each sample, quantified by the Bradford method^(^^34^^)^.

#### Western blotting

The protein concentration of the whole epididymal adipose tissue extract (including the cell membrane, cytoplasm and nucleus) was determined by the Bradford method^(^^34^^)^. Samples (25 µg of protein) were heated in Laemmli buffer at 100°C for 5 min, then loaded onto a 10 % SDS–polyacrylamide gel. Transfer to a nitrocellulose membrane was carried out at 4°C in the presence of methanol. Incubation with the primary antibodies (purchased from Santa Cruz Biotechnology) was performed overnight at 4°C in Tris-buffered saline solution containing Tween 20 (TBS-T) and 3 % non-fat dried milk. Antibody dilutions were: 1:200 for mouse anti-TLR-4 sc293072, 1:200 for mouse anti-β-actin sc47778, 1:100 for rabbit anti-phosphorylated NF-κB (ser536) sc33020, and 1:200 for mouse anti-total NF-κB sc8008. After incubation overnight at 4°C in TBS-T containing 1 % non-fat dried milk with the Abcam secondary antibodies (dilution 1:10 000) anti-rabbit ab97069 and anti-mouse ab98808, protein was revealed using the chemiluminescence method according to the manufacturer's instructions (ECL SuperSignal^®^ West Pico Chemiluminescent Substrate; Thermo Scientific). Band intensities were evaluated using Scion Image Software (Scion Corporation).

### Histological analysis

Adipose tissue was fixed in 4 % formaldehyde and embedded in paraffin. Two consecutive sections from each sample were cut (4 µm) and stained with haematoxylin/eosin. The entire slide was scanned using a 3DHISTECH Panoramic MIDI System attached to a Hitachi HV-F22 colour camera and ten fields/slide were analysed under 40× magnification in a blinded manner. The inflammatory reactions are reported as the number of inflammatory cells per high-power field. Using the same slides, the mean area of adipocytes was calculated using a method previously described by Osman *et al.* in 2013^(^^35^^)^.

### Statistical analysis

Results are expressed as means and standard deviations. Comparisons among groups were performed using two-way ANOVA for independent groups and were completed using Tukey's *post hoc* test. SigmaPlot 11.0 software (Systat Software Inc.) was used for statistical analyses. Differences were considered significant at *P* < 0·05. The statistical power for the main outcome variables was above 80 %.

## Results

### Body weight and body fat

There was no difference in energy intake between the C and HSF groups at any time (Table 1). The HSF caused changes in the body composition of the animals. At the end of 24 weeks, the HSF group had a greater average weight than the rats in the C24, HSF6 and HSF12 groups. The adiposity index was higher in all HSF groups compared with their respective controls, and in HSF12 and HSF24 compared with HSF6 (Table 1).

**Table 1.** Nutritional profile of the control diet (C) group and high-sugar/fat diet (HSF) group† (Mean values and standard deviations, *n* 8)

	Group														
	C6		HSF6		C12		HSF12		C24		HSF24				
Variables	Mean	sd	Mean	sd	Mean	sd	Mean	sd	Mean	sd	Mean	sd	*P* diet	*P* time	*P* interaction
IBW (g)	348·8	40·5	350·4	33·0	349·5	28·8	344·2	23·1	352·8	33·3	350·0	22·5	0·81	0·91	0·25
FBW (g)	456·0^A^	38·0	469·0^a^	40·3	499·3^A^	42·3	562·7^b^	54·0	568·4^B^	66·7	708·0*^c^	69·1	<0·001	<0·001	0·006
Weight gain (g)	107·2^A^	19·7	118·6^a^	32·0	149·9^A^	29·7	218·5*^b^	57·7	215·6^B^	58·7	358·0*^c^	72·1	<0·001	<0·001	0·002
Epididymal (g)	7·0^A^	1·8	10·8*^a^	3·2	8·9^A^	2·2	18·4*^b^	5·3	11·7^B^	4·4	26·7*^c^	7·4	<0·001	<0·001	0·7983
Retroperitoneal (g)	8·0^A^	2·6	15·6*^a^	5·3	10·7^B^	4·6	24·7*^b^	7·3	12·2^B^	4·7	26·7*^c^	7·4	<0·001	0·0010	0·6688
Visceral (g)	5·8^A^	1·8	9·9*^a^	3·4	8·1^B^	2·5	16·8*^b^	6·4	9·8^B^	3·7	24·0*^c^	5·7	<0·001	<0·001	0·9074
Total body fat (g)	20·9^A^	5·9	36·4*^a^	11·6	27·7^A^	8·5	59·9*^b^	17·4	36·6^A^	12·7	94·8*^c^	25·6	<0·001	<0·001	0·001
Adiposity index (%)	4·5^A^	1·0	7·6*^a^	1·8	5·5^A^	1·5	10·5*^b^	2·3	5·8^B^	1·7	10·9*^c^	2·3	<0·001	0·0029	0·9288
Food intake (g/d)	28·3	2·9	12·3*	2·0	27·7	2·2	12·7*	2·1	29·9	4·1	15·7*	1·4	<0·001	0·0060	0·6283
Water intake (ml/d)	35·8	5·0	27·9^A^	7·5	36·6	4·6	29·4^A^	8·8	45·3	5·3	43·2^B^	8·8	0·0064	<0·001	0·3913
Energy intake															
kcal/d	106·6	10·8	94·6	11·4	104·3	8·2	99·9	10·6	107·2	12·4	102·5	9·5	0·2032	0·4762	0·5623
kJ/d	446·0	45·2	395·8	47·7	436·4	34·3	418·0	44·4	448·5	51·9	428·9	39·7	0·2032	0·4762	0·5623

C6, control diet for 6 weeks; HSF6, high-sugar/fat diet for 6 weeks; C12, control diet for 12 weeks; HSF12, high-sugar/fat diet for 12 weeks; C24, control diet for 24 weeks; HSF24, high-sugar/fat diet for 24 weeks; IBW,  initial body weight; FBW, final body weight.

^A,B^ Mean values within a row with unlike uppercase letters were significantly different among the C groups (6 *v*. 12 *v*. 24 weeks) (*P* < 0·05).

^a,b,c^ Mean values within a row with unlike lowercase letters were significantly different among the HSF groups (6 *v*. 12 *v*. 24 weeks) (*P* < 0·05).

* Mean value was significantly different from that for the C group at the same time point (*P* < 0·05).

† Comparisons among groups were performed using two-way ANOVA for independent groups and were completed using Tukey's *post hoc* test.

### Plasma biochemical and hormonal measurements

An increase in TAG, glucose, insulin and leptin levels and a decreased level of adiponectin was detected in all animals in the HSF group compared with the C rats. However, when comparing animals subjected to the same diet for different periods of time, only leptin was increased in HSF12 and HSF24 rats compared with the HSF6 group. Animals that received HSF showed insulin resistance only at 24 weeks compared with control animals characterised by increased HOMA-IR (Table 2). There was no difference in NEFA levels among the groups.

**Table 2.** Plasma biochemical and hormonal measurements† (Mean values and standard deviations, *n* 8)

	Groups														
	C6		HSF6		C12		HSF12		C24		HSF24				
Variables	Mean	sd	Mean	sd	Mean	sd	Mean	sd	Mean	sd	Mean	sd	*P* diet	*P* time	*P* interaction
TAG (mmol/l)	0·47^A,B^	0·13	1·03*^a,b^	0·32	0·43^A^	0·04	0·93*^a^	0·25	0·58^B^	0·17	1·14*^b^	0·25	<0·001	0·0150	0·2970
NEFA (mmol/l)	0·3	0·1	0·3	0·1	0·4	0·1	0·4	0·1	0·3	0·1	0·4	0·1	0·0192	0·0515	0·4190
Glucose (mmol/l)	4·77	0·58	5·68*	0·40	6·53	1·17	7·77*	1·39	5·38	0·04	6·51*	0·71	<0·001	0·0069	0·7055
Insulin (ng/ml)	1·9	0·8	3·9*	1·6	2·7	2·1	4·2*	1·3	2·4	0·8	5·4*	1·6	<0·001	0·1487	0·3074
HOMA-IR	0·9	0·5	3·8	2·5	2·5	4·4	4·2	2·4	1·4	1·1	7·0*	4·8	<0·001	0·232	0·194
Leptin (ng/ml)	2·5^A^	0·8	6·2*^a^	0·8	2·8^A,B^	0·7	11·4*^b^	5·8	3·7^B^	2·4	13·9*^b^	3·2	<0·001	0·0053	0·5502
Adiponectin (ng/ml)	19·4	7·2	11·4*	1·6	20·1	4·4	11·7*	2·6	19·0	4·0	13·1*	1·6	<0·001	0·724	0·370

C6, control diet for 6 weeks; HSF6, high-sugar/fat diet for 6 weeks; C12, control diet for 12 weeks; HSF12, high-sugar/fat diet for 12 weeks; C24, control diet for 24 weeks; HSF24, high-sugar/fat diet for 24 weeks; HOMA-IR, homeostasis model assessment.

^A,B^ Mean values within a row with unlike uppercase letters were significantly different among the C groups (6 *v*. 12 *v*. 24 weeks) (*P* < 0·05).

^a,b^ Mean values within a row with unlike lowercase letters were significantly different among the HSF groups (6 *v*. 12 *v*. 24 weeks) (*P* < 0·05).

* Mean value was significantly different from that for the C group at the same time point (*P* < 0·05).

† Comparisons among groups were performed using two-way ANOVA for independent groups and were completed using Tukey's *post hoc* test.

### Adipose tissue and serum adipokine measurements

**Fig. 1.**
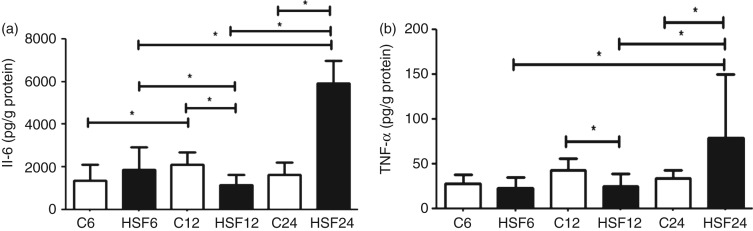
Cytokine levels (pg/g protein) in epididymal adipose tissue in control diet (C) and high-sugar/fat diet (HSF) groups over 6, 12 and 24 weeks (*n* 8 animals/group). (a) IL-6 level; (b) TNF-α level. Values are means, with standard deviations represented by vertical bars. * Mean values were significantly different (*P* < 0·05).

No differences were found in the plasma levels of TNF-α and IL-6 when comparing different diets or different durations (data not shown). In adipose tissue, animals in the HSF24 group exhibited elevated levels of these cytokines compared with the C24 group, as well as the HSF6 and HSF12 groups (Fig. 1). However, HSF12 had lower levels of TNF-α and IL-6 compared with C12.

### Adipose tissue area

**Fig. 2.**
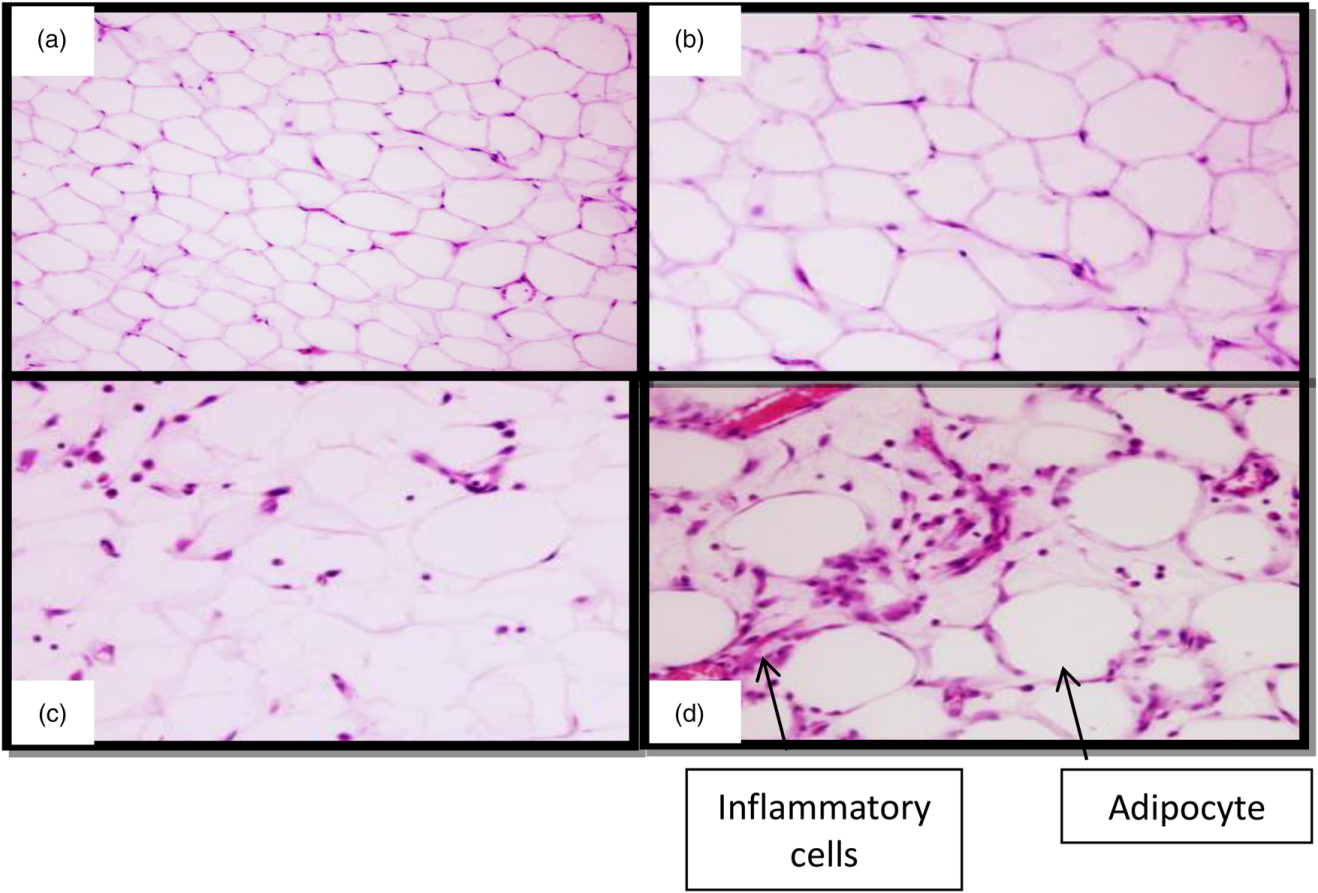
Inflammatory cells in adipose tissue. (a) Control group; (b) group fed high-sugar/fat diet for 6 weeks (HSF6); (c) group fed high-sugar/fat diet for 12 weeks (HSF12); (d) group fed high-sugar/fat diet for 24 weeks (HSF24) (*n* 8 animals/group). 40× Magnification.

Table 3 shows the mean area of adipocytes in control animals and those fed the HSF in the three periods of the experiment. Note that there was an increase in the area of the adipocytes in animals fed the HSF only at 24 weeks compared with the C24, HSF6 and HSF12 groups. Fig. 2 shows images taken for the assessment of inflammatory cell infiltration. A greater number of cells was observed in the HSF24 group.

**Table 3.** Mean area of adipocytes in epididymal adipose tissue of control diet (C) and high-sugar/fat diet (HSF) groups† (Mean values and standard deviations, *n* 8)

	Area of adipocytes (mm^2^)					
Group	Mean	sd	Number of inflammatory cells/field	*P* diet <0·001	*P* time <0·001	*P* interaction 0·004
C6	1·0^A^	0·2	1–5			
HSF6	1·4^a^	0·2	1–5			
C12	1·1^A^	0·4	1–5			
HSF12	1·4^a^	0·1	20–40			
C24	2·8^B^	1·2	5–20			
HSF24	5·0*^b^	0·9	>40			

C6, control diet for 6 weeks; HSF6, high-sugar/fat diet for 6 weeks; C12, control diet for 12 weeks; HSF12, high-sugar/fat diet for 12 weeks; C24, control diet for 24 weeks; HSF24, high-sugar/fat diet for 24 weeks.

^A,B^ Mean values within a column with unlike uppercase letters were significantly different among the C groups (6 *v*. 12 *v*. 24 weeks) (*P* < 0·05).

^a,b^ Mean values within a column with unlike lowercase letters were significantly different among the HSF groups (6 *v*. 12 *v*. 24 weeks) (*P* < 0·05).

* Mean value was significantly different from that for the C group at the same time point (*P* < 0·05).

† Comparisons among groups were performed using two-way ANOVA for independent groups and were completed using Tukey's *post hoc* test.

### Western blotting

**Fig. 3.**
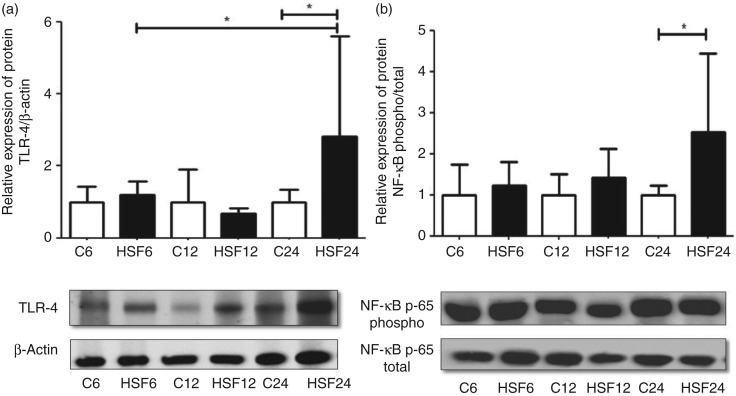
Relative expression of protein in epididymal adipose tissue in control diet (C) and high-sugar/fat diet (HSF) groups over 6, 12 and 24 weeks (*n* 8 animals/group). (a) Toll-like receptor-4 (TLR-4); (b) NF-κB. Values are means, with standard deviations represented by vertical bars. * Mean values were significantly different (*P* < 0·05).

Fig. 3 shows the protein expression of TLR-4 and NF-κB in epididymal adipose tissue. At the end of 24 weeks, the animals in the HSF24 group showed higher TLR-4 expression than those in the C24 and HSF6 groups. Similar to TLR-4, the expression of NF-κB increased in epididymal adipose tissue after 24 weeks in the HSF group when compared with animals in the C24 group.

## Discussion

In the present study using Wistar male rats fed an HSF, we evaluated the temporal relationship between the manifestation of metabolic parameters and the effects of inflammation in adipose tissue via TLR-4. According to the WHO, obesity is defined as an excessive accumulation of body fat^(^^36^^)^. In 2014, Strissel *et al*.^(^^37^^)^ associated obesity with chronic low-grade inflammation, called metainflammation^(^^38^^)^, which differs from the classic inflammatory response against injury or pathogens^(^^39^^)^. Chronic consumption of an HSF is associated with metabolic changes, since it triggers an increase in body weight and obesity^(^^9^^)^. These factors are associated with high blood pressure, as well as biochemical and hormonal changes, such as increased blood glucose, TAG, NEFA, leptin and insulin, as well as reduced adiponectin^(^^40^^,^^41^^)^. However, it is still unclear how the duration of this diet is related to metabolic changes and whether these changes occur before or after inflammation.

HOMA-IR has been used to assess overall insulin sensitivity in human subjects and rats with different degrees of insulin sensitivity^(^^42^^)^. Therefore, HOMA-IR is a good predictor of whole-body insulin sensitivity. In our study, the modification of parameters including glucose, TAG, leptin and insulin, as well as low adiponectin, started as early as 6 weeks on the diet. There was also a greater adiposity index observed in these animals. All these conditions persisted up to 24 weeks, even without higher energy intake. This underscores the notion that diet components are an important factor in the manifestation of the MS. The HSF is a combination of palatable foods with high energy density, reflecting the Western dietary pattern. These data corroborate the HSF as a trigger of metabolic and hormonal changes, abdominal circumference, and expansion of fat mass^(^^43^^,^^44^^)^. More specifically, a higher proportion of SFA (myristic (C14), palmitic (C16) and stearic (C18)) in relation to unsaturated fats (mono- and polyunsaturated) is associated with high adiposity and central fat deposit^(^^45^^)^. High levels of carbohydrates are also able to mobilise fat from the periphery to central deposits and reduce the activity of adiponectin in peripheral tissues^(^^46^^)^. When there is an expansion of fat mass, there is also adipocyte hypertrophy, which is responsible for the production of adipokines, including TNF-α and IL-6^(^^47^^)^. In our work, at 6 and 12 weeks, although the animals presented an increase in the adiposity index, hypertrophy was not present and was only observed at 24 weeks, accompanied by inflammation and increased TNF-α and IL-6 levels. Therefore, the inflammatory state was observed only together with adipocyte hypertrophy, suggesting that adipose tissue is made up of mature and immature adipocytes (low fat content). Under excessive energy supply conditions, immature adipocytes would be responsible for the accumulation of fat, giving the adipose tissue a uniform appearance that still does not characterise hypertrophy.

Two distinct macrophage populations can be identified in adipose tissue: M1 and M2^(^^48^^)^. The M1 profile produces proinflammatory cytokines that affect cell proliferation and promote insulin resistance, while the M2 population is associated with an anti-inflammatory phenotype that protects against metabolic disorders^(^^49^^,^^50^^)^. Lean individuals express a balance in the M1/M2 profile in adipose tissue, while obese individuals initially show an increase in the M2 profile, a defence mechanism to combat possible inflammation^(^^50^^)^. Our data show that, at 12 weeks, the animals showed a reduction in TNF-α and IL-6, providing evidence for this compensatory response in adipose tissue. This mechanism can occur to reduce inflammation and metabolic deterioration of the tissue. However, at 24 weeks, due to the hypertrophy of adipocytes and increased body fat, a shift may have occurred in the profile of these macrophages towards M1, causing an increase in proinflammatory cytokines. Corroborating this hypothesis, a murine study by Shaul *et al*.^(^^51^^)^ also showed an enhanced M2 phenotype in adipose tissue in obese mice after 12 weeks on a high-fat diet compared with mice fed the same diet for 8 weeks.

Increased adiposity is associated with the activation and migration of inflammatory cells into the adipose tissue, as well as proinflammatory cytokine secretion and development of low-grade chronic inflammation^(^^52^^)^. Besides the hypertrophy of adipose tissue and macrophage profile in tissue, another factor that can enhance this inflammatory condition is the activation of TLR-4 receptors^(^^19^^)^. Our results show an increase in expression of adipocytes at 24 weeks. The literature shows that this activation can occur by an increased release of fatty acids by adipose tissue, by SFA intake^(^^51^^)^ or by the change in intestinal flora and lipopolysaccharide stimulus^(^^53^^)^. In the present study, we can attribute the increase in TLR-4 to the dietary fat stimulus, since elevated NEFA in circulation were not found. TLR-4 activates the transcription factor NF-κB, leading to increased production of proinflammatory cytokines^(^^54^^)^, reinforcing the importance of this pathway.

In obesity, the degree of inflammation correlates with the extent of insulin resistance, a mechanism involving TNF-α, which interferes with the phosphorylation of the insulin receptor, impairing its glucose uptake function^(^^55^^)^. Our work shows that insulin resistance is present, together with the inflammatory response, in adipose tissue after 24 weeks, along with adipocyte hypertrophy. Thus, these data allow us to hypothesise that insulin resistance is influenced by inflammation of the adipose tissue, which in turn is associated with adipocyte hypertrophy and TLR-4 activation. We can conclude that the MS occurs independently of inflammation in adipose tissue and that inflammation is associated with adipocyte hypertrophy, which varies according to the duration of exposure to an HSF.

### Final considerations

Although the present study was carried out in rats, the mechanisms related to the development of the MS and inflammation may be similar to those involved in clinical obesity, since after the onset of inflammation in adipose tissue, dependent on adipocyte hypertrophy, the organism may develop new co-morbidities over time, and potentiate the pre-existing ones. The results of the present study are clinically important because they provide information that may promote interventions to prevent adipocyte hypertrophy and inflammation, being a preventive measure in the development of co-morbidities arising from this process.
